# Cognitive and Mental Health Predictors of Withdrawal Severity During an Active Attempt to Cut Down Cannabis Use

**DOI:** 10.3389/fpsyt.2018.00301

**Published:** 2018-07-11

**Authors:** Janna Cousijn, A. C. K. van Duijvenvoorde

**Affiliations:** ^1^Addiction Development and Psychopathology Lab, Department of Psychology, University of Amsterdam, Amsterdam, Netherlands; ^2^Brain and Development Lab, Department of Psychology, Leiden University, Leiden, Netherlands; ^3^Leiden Institute for Brain and Cognition, Leiden, Netherlands

**Keywords:** cannabis, cognitive control, cannabis use disorder, craving, depression, withdrawal

## Abstract

A milestone in cannabis research is the establishment of a clinically relevant cannabis withdrawal syndrome, yet little is known about the underlying mechanisms. We investigated the predictive role of mental health and cognitive factors in withdrawal severity during an active attempt to cut down, relative to uninterrupted cannabis use. Ninety heavy cannabis users were randomly assigned to an experimental or control group. The experimental group was asked to cut down substance use for 1 week. Past week substance use, substance use-related problems, depressive symptoms, cravings, and cognitive control were assessed at baseline. Past week substance use and withdrawal severity were assessed at follow-up. The experimental group reduced their cannabis use more and experienced more withdrawal than the control group. Hierarchical regression analysis per predictor indicated that cannabis use-related problems, depressive symptoms, and cannabis craving, but not cognitive control, predicted stronger withdrawal. Craving uniquely predicted withdrawal in the experimental group. A combined hierarchical regression indicated that only depressive symptoms and cannabis use-related problems uniquely predicted withdrawal across groups. These results suggest that depressive symptoms and cannabis use-related problems are generally indicative of cannabis withdrawal severity, whereas craving specifically predicts cannabis withdrawal during an active attempt to cut-down cannabis use.

## Introduction

One of the milestones in cannabis research is the establishment of a clinically relevant cannabis withdrawal syndrome (1). As such, cannabis withdrawal is a new diagnostic criterion for cannabis use disorders (CUDs) in the latest edition of the diagnostic statistical manual [DSM-5; (2)]. Cannabis withdrawal refers to mental (e.g., mood swings, sleep disruptions) and physical (e.g., headaches, nausea) discomfort that cannabis users may experience after discontinuation or reductions in use. Supporting the maintenance of addictive behaviors, poor cognitive functioning and mental health may play an important role in the severity of mental discomfort during withdrawal. However, little is still known about the underlying mechanisms. We therefore assessed the role of both cognitive and mental health factors in the subjective severity of cannabis withdrawal during an active attempt to cut down cannabis use in heavy cannabis users.

Given the recognized role of withdrawal in relapse, withdrawal reduction was one of the primary targets for the development of pharmacotherapies for substance use disorders (SUDs) over the past decades (3). The existence of withdrawal after discontinuation of cannabis use has long been questioned, paralleling the lack of pharmacotherapies approved for treating CUDs (4). However, the past decades, many studies confirmed withdrawal is common in both treatment and non-treatment seeking cannabis users [for reviews see (5, 6)]. Moreover, there appears to be a bidirectional relationship between withdrawal and dependence severity; That is, higher CUD severity has been found to predict more severe withdrawal [e.g., (7, 8); but see (9)] and more severe withdrawal has been found to predict more severe future dependence (10). It has also repeatedly been shown that cannabis users who experience more severe withdrawal during a quit attempt reinitiate use sooner (8, 11–13). Finally, withdrawal has been found to be higher in individuals with more severe depressive symptoms (7, 14).

Although not all users will experience withdrawal (9), cannabis withdrawal generally peaks within the first week of abstinence and predicts relapse (8, 13, 15). Most previous studies specifically investigated the mechanisms underlying cannabis withdrawal during an attempt to remain abstinent. However, daily fluctuations in withdrawal symptoms are thought to play a prominent role in non-abstinent users as well; The acute withdrawal symptoms that emerge after using induce a state of negative affect that, in turn, increases the motivation to use again (16, 17). In line with this, using frequent daily withdrawal assessments in heavy non-abstinent cannabis users, it has been shown that cannabis withdrawal increases just prior to cannabis use (18). As such, to help us understand who is at a greater risk of relapse after abstinence, an important next step is to reveal predictors of withdrawal severity during an active attempt to cut-down cannabis use *compared to* uninterrupted cannabis use.

Theoretical models of addiction suggest that, beside withdrawal severity, poor cognitive control over the extremely strong motivations to use (e.g., craving) is thought to play a key role in the development and maintenance of SUDs (16, 19–23). Few studies specifically investigated the role of craving and cognitive control in CUDs, however, there is preliminary evidence that both processes play a prominent role in the development of CUDs and relapse. Craving is a complex multifaceted process that represents the extreme urge to use drugs commonly experienced during abstinence (17). Exposure to cannabis-related cues can also induce craving in individuals with a CUD (24, 25) and craving can predict treatment outcomes in adolescents with a CUD (26). Regarding cognitive control, cannabis intoxication can temporarily impair planning, organizing, problem solving, decision-making, memory, and emotional control (27). Although contradictory findings have been reported, CUDs are associated with similar impairments in cognitive control (28, 29) and withdrawal during abstinence parallels a temporarily (further) decline (30, 31). Neural activity in brain areas involved in cognitive control may predict escalation of cannabis use in heavy cannabis users; Using the Iowa Gabling Task, higher win-related activity in the superior frontal gyrus and higher activity during the anticipation of disadvantageous decisions in the frontal pole were found to be associated with an increase in weekly cannabis use 6 months later (32). Similarly, using the N-Back working-memory task, functionality of the fronto-parietal executive network also predicted changes in cannabis use (33). Interestingly, better cognitive control as measured with the classical Stroop (34) has been found to relate to less cannabis cue-induced craving in heavy cannabis users (35). Further supporting the link between cognitive control and craving, higher neural activity in brain areas involved in cognitive control in response to watching cannabis cues related to less craving in heavy cannabis users (36).

Although the association between withdrawal severity, mental health problems and treatment outcomes is evident (6–9), the association between cognition and cannabis withdrawal severity is unclear. The above described preliminary findings of the association between cognition and cannabis use suggest that cognitive factors including craving and cognitive control, as well the severity of CUD-related mental health problems like cannabis use-related problems and depression can predict withdrawal severity during an active attempt to cut down or quit cannabis use. More specifically, better cognitive control may help in actively reducing the motivation to use cannabis and mental discomfort from withdrawal, and withdrawal may temporarily lower cognitive control. In turn, higher baseline craving and, as suggested by previous studies (7, 8, 14), especially severity of depressive symptoms and CUD may increase the likelihood to experience withdrawal.

To test this hypothesis, craving, cognitive control [Classical Stroop (34) and Colombia Card Test (37)], substance use-related problems (cannabis, nicotine, alcohol), and symptoms of depression were assessed in a large group of almost daily cannabis users during a baseline laboratory test session. Given the presumed role of cannabis withdrawal severity in continued daily cannabis use, as well as relapse after abstinence, it is important to study predictors of withdrawal in cannabis users who try to lower their use compared to those who continue their use. To specifically investigate withdrawal during an active attempt to cut down vs. uninterrupted cannabis use, the heavy cannabis users were randomly assigned to an experimental or control group. The experimental group was explicitly instructed to use as little cannabis and other substances during following week. Substance use and withdrawal severity of that week were subsequently assessed through a telephone interview exactly 1 week later. Given the multifaceted complex nature of cognitive control and the relevance of emotional control for addiction, both a relatively cold, emotion devoid, general executive functioning task (Stroop) and a relatively hot emotional decision making task (CCT) were included in the design. We expected (i) baseline craving, cannabis use-related problems, and symptoms of depression to be positively related to more severe withdrawal, and (ii) cognitive control to be negatively related to more severe withdrawal.

## Materials and methods

### Participants

Ninety-three heavy cannabis users (21 females; 18–31 years) were recruited at educational institutions and in Dutch cannabis outlets. Heavy cannabis use was defined as smoking cannabis more than 3 days a week for at least 1 year and having a Cannabis Use Disorder Identification Test-Revised [CUDIT-R; (38)] score of ≥10. Approximately 95% percent of the cannabis users that score 10 or more on the CUDIT-R have a CUD (38). Potential participants were excluded if they currently used psychotropic medication or had a treatment history for any psychiatric disorder. All participants signed informed consents and the local Ethics Committee of Leiden University approved all procedures in this study. Of the 93 participants, three were excluded from further analyses due to technical problems. The final sample thus included 90 heavy cannabis users (Table 1).

**Table 1:** Medians (SD) of sample characteristics and study measures in all participants at baseline (Complete sample), and for the participants that remained in the study at follow-up (Control and Experimental Group, excluding drop-out).

	**Complete sample (*n* = 90)**	**Control (*****n*** = **36, excluding drop-out)**		**Experimental (*****n*** = **29, excluding drop-out)**	

	**Baseline**	**Baseline**	**Follow-up**	**Baseline**	**Follow-up**
Female (%)	23	19		24	
Age (years)	21.4 (18.3–28.3)	20.9 (18.3–25.7)		21.4 (18.3–28.3)	
Cannabis use:					
Days per week	6 (3–7)	6 (3–7)		6 (3-7)	
TLFB, grams past week	2.7 (0.7–16.8)	4.3 (1.8–15.7)	3.2 (0.3–11.8)	5.2 (0.7–16.8)	2.4 (0–10)
CUDIT-R	17 (10–27)	16 (10–26)		17 (11–27)	
Mixed joints with tobacco (%)	91	89		90	
Alcohol use:					
TLFB, glasses past week	10 (0–55.2)	11 (0–51.5)	12 (0–37)	10 (0–55.2)	4 (0–100)
AUDIT	11.5 (0–28)	11 (3–24)		12 (0–28)	
Smoking:					
Smokers (%)	72	64		72	
TLFB, cigarettes past week	42.5 (0–161)	42 (0–160)	32 (0–195)	47 (0–161)	42 (0–160)
FTND	1 (0–7)	0 (0–7)		2 (0–7)	
Depression (BDI)	7 (0–30)	5 (0–30)		8 (2–26)	
Cognitive factors:					
Cannabis craving (MCQ-SF)	3.2 (1.5–5.5)	3.2 (1.5–5.2)		3.2 (1.7–5.5)	
Stroop Interference score (ms)	30.2 (11.7–54.1)	27.5 (11.7–54.1)		31.8 (13.8–52)	
CCT n cards	9.6 (2.7–15)	8.5^*^ (2.7–13.9)		10^*^ (5.8–15)	
Cannabis withdrawal at follow-up (MWC)	–	–	8^*^ (0–21)	–	11^*^ (0–36)

*Range is reported between brackets. TLFB, Timeline Follow Back; CUDIT-R, Cannabis Use Disorder Identification Test-Revised; AUDIT, Alcohol Use Disorder Identification Test; BDI, Beck Depression Inventory; FTND, Fagerstrom Test for Nicotine Dependence; MCQ-SF, Marihuana Craving Questionnaire-Short Form; CCT, Columbia Card Task; MWC, Marihuana Withdrawal Checklist*.

* *significant group differences at p < 0.05*.

### Questionnaires on substance use, craving, and psychological functioning

Cannabis craving was assessed with the 12-item Marijuana Craving Questionnaire-Short Form [MCQ-SF; (39)] at the start of the baseline test-session. The MCQ-SF assesses various dimensions of craving (e.g., expected positive outcomes, desire to use and intention to use) on a 7-point scale ranging from “strongly disagree” (1) to “strongly agree” (7). Total craving score was obtained by averaging all item scores. The Timeline Follow-Back (TLFB) was used to measure cannabis, alcohol, cigarette and other substance use during the past 7 days at baseline and follow-up (40). The 8-item CUDIT-R was used to measure severity of cannabis use-related problems during the past 6 months at baseline (38). The CUDIT-R assesses the frequency of cannabis use, symptoms of dependence and other psychological problems, scaled from never (0) to almost daily (4). Total CUDIT-R score was obtained by summing all item scores. The 10-item Alcohol Use Disorder Identification Test (AUDIT) was used to measure severity of alcohol use-related problems during the past 6 months at baseline (41). Moreover, the 6-item Fagerstrom Test for Nicotine Dependence (FTND) was used to assess severity of nicotine dependence during the past 6 months at baseline (42). The 21-item Beck Depression Inventory (BDI) was used to measure symptoms of depression at baseline (43). Finally, the 15-item Marijuana Withdrawal Checklist (MWC) was used to assess cannabis withdrawal symptom severity during the past week at follow-up (15). This questionnaire assesses mental (e.g., depressed mood, irritability, aggression) and physical (e.g., headaches, shakiness) symptoms commonly associated with cannabis withdrawal. Participants can also add additional symptoms they experienced. Participants rated the severity of each symptom on a 4-point scale from “not at all” (0) to “severe” (3). Total withdrawal severity was obtained by summing severity ratings.

### Cognitive control tasks

The validated Dutch paper version of the Classical Stroop Task (34) and the computerized “hot” Columbia Card Task [CCT; (37)] were adminstered. During the Stroop, participants first read aloud color words (i.e., blue, green, red, yellow) as fast as possible printed in black ink, then named aloud colors of solid colored patches, and finally read aloud color words printed in an incongruent color (e.g., red printed in blue). The difference between the congruent (first two) and incongruent (last) subtasks was taken as a measure of cognitive control, with high scores indicating more interference and therefore lower cognitive control.

The CCT is a computerized card game with win and loss cards during which participants are instructed to earn as much points as possible. Each game round participants view 32 faced-down cards and turn as many as they want before deciding to stop the current round and cash the accumulated points. However, if they turn a loss card they will lose points and the game round ends. Points gained with a win card (10 or 30), points lost with a loss card (250 or 750), and the number of loss cards (1 or 3) varied. The average number of turned cards across 24 game rounds was used in subsequent analyses. The CCT was made incentive compatible by instructing participants that their scores of three randomly drawn rounds would be averaged and summed together. This total score was used in a weighted lottery procedure (see *Procedure* below).

### Experimental manipulation

The experimental group received the following instruction: “*For the coming week, please lower your cannabis, nicotine, alcohol and other drug use as much as possible. Note that each joint, cigarette or beer you use less is incredibly good. In a week I will call you to ask how this went; this conversation will take approximately 15 min. In addition, we raffle 50 Euro among everyone who takes part in this follow-up interview, irrespective of your cannabis, and other drug use*.” The control group was only told that they would be contacted for a follow-up interview regarding their cannabis, and other drug use, and that we raffled 50 Euros among the follow-up participants.

### Procedure

The experiment consisted of a baseline test-session and a telephone follow-up interview. During the baseline test-session participants first completed the MCQ-SF, followed by the Stroop and CCT on a laptop. The remaining questionnaires were filled out after the tasks in the following order; TLFB, CUDIT-R, AUDIT, FTND, and BDI. The baseline test-session took approximately 45 min for which each participant received 7.50 Euro. Exactly 1 week after the test session, all participants were contacted again for a short telephone interview that contained first the TLFB followed by the MWC. A shopping voucher of 50 Euro was raffled among participants who completed the follow-up interview. Participants were instructed that this lottery was weighted upon their CCT score, with higher scores relating to more chance to gain the 50 Euro voucher.

### Statistical methods

To assess sample characteristics and change in substance use between the experimental and control group, independent sample *t*-tests and repeated measures ANOVAs were used. Although some of our variables are relatively skewed, these tests are described as robust with respect to the assumption of normality. This means that some deviation away from normality does not have a large influence on Type I error rates, particularly when group sizes are moderate to large and relatively equally sized (44). When the equality of variance assumption was violated, we report corrected degrees of freedom and corrected *t*-values. Finally, we used hierarchical regression analyses were used for testing predictors of withdrawal. Inspection of residual probability plots indicated no violation of assumptions of normality, linearity, and heteroscedasticity, nor were there influential cases detected (maximum Cook's distance = 0.347).

## Results

### Sample characteristics at baseline and at 1-week follow-up

Attrition at follow-up was 28% and did not significantly differ between the experimental (*n* = 17) and control (*n* = 8) group [χ_(1)_ = 3.2, *p* = 0.072]. Participants that dropped out differed on none of the baseline measures from the participants that remained in the study as indexed by independent sample *t*-test comparisons (all *p*-values > 0.1), except on the CUDIT-R, which was higher in the participants that dropped out [*t*_(88)_ = −2.2, *p* = 0.032]. All subsequent analyses were conducted excluding the participants who dropped out at follow-up.

**Figure 1:**
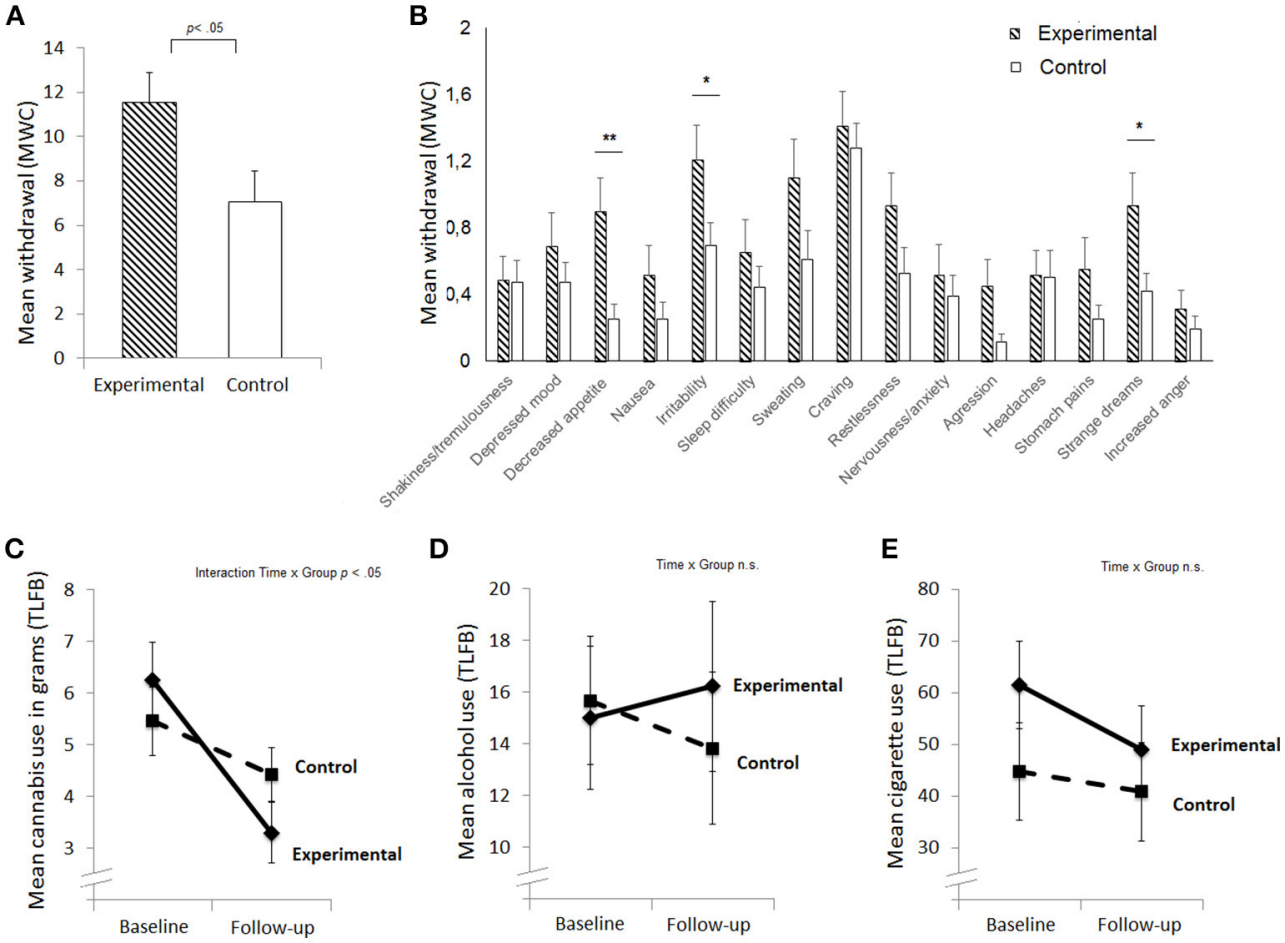
Mean levels of withdrawal **(A)**, mean withdrawal score per symptom **(B)**, cannabis use in the past week **(C)**, alcohol use in the past week in standard units **(D)**, and smoking in the past week in number of cigarettes **(E)** at baseline and follow-up for the experimental and control group. Standard errors are plotted; ^**^p < 0.01, ^*^p < 0.05; TLFB, Timeline Follow Back; MWC, Marijuana Withdrawal Checklist.

Independent *t*-tests were performed to test whether the experimental and control group differed significantly on any of the baseline measures. Results showed that the experimental and control groups did not differ significantly in any of the baseline measures, except CCT score, in which the experimental group (*M* = 10.2, *SD* = 2.0) sampled more cards than the control group (*M* = 8.6, *SD* = 2.4), *t*_(63)_ = 2.99, *p* = 0.004. As expected, the experimental group reported higher withdrawal at follow-up than the control group [*t*_(43.2)_ = 2.34, *p* = 0.024, Cohen's *d* = 0.63; Figure 1A]. Exploratory analyses of individual withdrawal symptoms indicated that group differences were most apparent in physical [decreased appetite; *t*_(39.7)_ = 2.93, *p* = 0.006] and mental symptoms such as increased irritability [*t*_(50.2)_ = 2.07, *p* = 0.037], and strange dreams [*t*_(44.1)_ = 2.28, *p* = 0.028], see Figure 1B.

Next we aimed to assess if groups differed in their cannabis use over time (Figures 1C–E). A repeated measure ANOVA was performed with used grams per week (TLFB) at baseline and follow-up as within-subject factor and group as between-subject factor. Cannabis use was lower at follow-up [main effect Time: *F*_(1, 63)_ = 29.7, *p* < 0.001, partial η^2^ = 0.32], and this decline was larger for the experimental group [interaction effect Time x Group: *F*_(1, 63)_ = 6.9, *p* = 0.011, partial η^2^ = 0.1; see Figure 1C], indicating that participants indeed engaged in an active attempt to cut down. Alcohol use did not differ between sessions, nor was there an interaction effect between Time and Group (*p*s > 0.3). Cigarette use was slightly lower at follow-up independently of group [main effect Time: *F*_(1, 63)_ = 4.4, *p* = 0.04, partial η^2^ = 0.07; Figure 1E]. The absolute decline in cannabis (*r* = −0.215, *p* = 0.09), cigarette (*r* = −0.030, *p* = 0.84), and alcohol use (*r* = 0.046, *p* = 0.72) did not significantly correlate with cannabis withdrawal.

Cannabis, cigarette, and alcohol use are highly comorbid in the current sample (Table 1). Pairwise correlations were computed to investigate the association between cannabis, cigarette, and alcohol use (baseline TLFB, follow-up TLFB, change in TLFB) and problem behavior related to substance use (CUDIT-R, FTND, AUDIT). Cannabis use did not correlate with cigarette (baseline: *r* = 0.132, *p* = 0.295; follow-up: *r* = 0.025, *p* = 0.845) and alcohol use (baseline: *r* = 0.050, *p* = 0.691; follow-up: *r* = 0.016, *p* = 0.902). Similarly, change in cannabis use did not correlate with change in cigarette (*r* = 0.121, *p* = 0.409) and alcohol use (*r* = 0.180, *p* = 0.173). Correlations between measures of problem behaviors showed that the CUDIT-R correlated significantly with the FTND (*r* = 0.363, *p* = 0.003), but not with the AUDIT (*r* = 0.119, *p* = 0.344).

### Predictors of withdrawal

**Figure 2:**
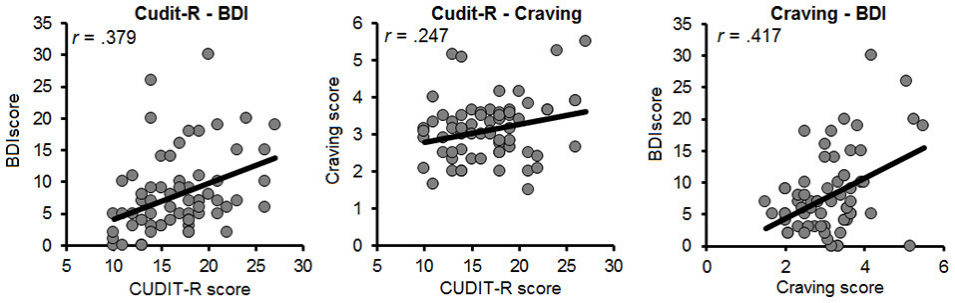
Scatterplots for the relation between indices of mental-health factors **(Left)**, and craving and mental-health factors **(Middle and Right)**. BDI, Beck Depression Inventory; CUDIT-R, Cannabis Use Disorder Identification Test-Revised; MCQ, Marijuana Craving Questionnaire.

Pairwise correlations were computed between all predictors in the participants who completed the entire study (Figure 2). The BDI was moderately correlated with the CUDIT-R (*r* = 0.379, *p* = 0.002) and craving (*r* = 0.417, *p* = 0.001). Moreover, craving was weakly correlated with the CUDIT-R (*r* = 0.247*, p* = 0.047). The two cognitive control measures did not significantly correlate with each other (Stroop-CCT *r* = 0.211, *p* = 0.091), with mental health (Stroop-BDI *r* = 0.047, *p* = 0.709; Stroop-CUDIT-R *r* = 0.051, *p* = 0.685; CCT-BDI *r* = 0.215, *p* = 0.085; CCT-CUDIT-R *r* = 0.089, *p* = 0.480), and with craving (Stroop-craving *r* = 0.077, *p* = 0.543; CCT-craving *r* = 0.015, *p* = 0.904).

**Figure 3:**
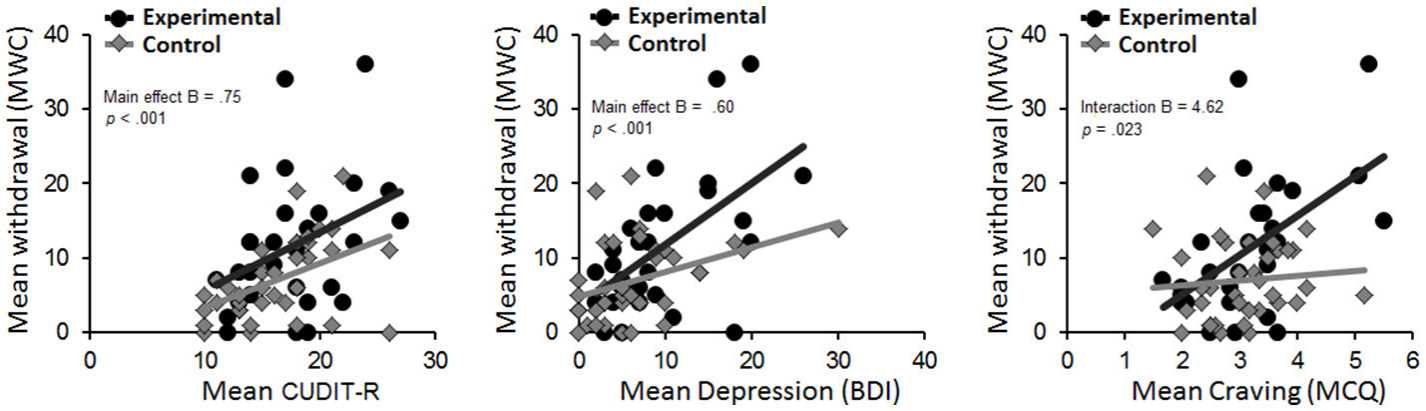
Scatterplots for the relation between level of withdrawal at follow-up and CUDIT-R **(Left)**, Depression **(Middle)**, and Craving **(Right)** score at baseline for the experimental and control group separately. BDI, Beck Depression Inventory; CUDIT-R, Cannabis Use Disorder Identification Test-Revised; MCQ, Marijuana Craving Questionnaire.

A first aim was to assess if mental health, craving and cognitive control predicted withdrawal severity (Figure 3). A second aim was to assess if these effects were specific for an active attempt to cut-down substance use. A hierarchical regression analysis was performed per predictor of interest. For each of these regressions, MWC score was the dependent variable. The main effect of the predictor under study was entered first, after which a main effect of group was entered in the second step, and finally an interaction effect between group and predictor was added.

Higher CUDIT-R score was a significant predictor of withdrawal (*B* = 0.75, *SE* = 0.2, *p* < 0.001, *R*^2^ = 18.1%). Adding the main effect of group explained significantly more variance [*F*_change(1, 62)_ = 4.37, *p* = 0.04, *R*^2^ = 23.5%], although there was no significant group x CUDIT-R interaction (*p* = 0.64).

Higher BDI score also was a significant predictor of withdrawal (*B* = 0.60, *SE* = 0.13, *p* < 0.001, *R*^2^ = 26%). Adding group (*B* = −2.85, *SE* = 1.65, *p* = 0.089) as well as the group x BDI interaction (*B* = 0.483, *SE* = 0.25, *p* = 0.062) did not explain significantly more variance.

MCQ-SF score was a significant predictor of withdrawal (*B* = 3.41, *SE* = 1.05, *p* = 0.002, *R*^2^ = 14.4%), indicating that greater craving was predictive of greater cannabis withdrawal. Adding the main effect of group [*F*_change(1, 62)_ = 5.7, *p* = 0.02, *R*^2^ = 21.6%], as well as a group x craving interaction [*F*_change(1, 61)_ = 5.5, *p* = 0.023, *R*^2^ = 28.1%] explained significantly more variance. Follow-up tests of this interaction term indicated that greater craving predicted higher withdrawal symptoms in the experimental (*B* = 5.27, *SE* = 1.55, *p* = 0.002), but not in the control group (*B* = 0.64, SE = 1.22, *p* = 0.6).

Finally, cognitive control measures did not significantly predict withdrawal symptoms in any of the models. That is, CCT was not a significant predictor of withdrawal (*B* = 0.317, *SE* = 0.389, *p* = 0.107). While there was a main effect of group [*F*_change(1, 62)_ = −4.49, *p* = 0.025], there was no group x CCT interaction (*B* = −0.144, *SE* = 0.852, *p* = 0.866). Similarly, Stroop was not a significant predictor of withdrawal (*B* = 0.054, *SE* = 0.084, *p* = 0.522). While there was a main effect of group [*F*_change(1, 62)_ = 5.69, *p* = 0.02], there was again no group x Stroop interaction (*B* = −0.012, *SE* = 0.166, *p* = 0.942).

Given the significant correlations between BDI, CUDIT-R and craving, a *combined* hierarchical regression was performed to assess the unique contribution of these significant predictors of interest. Following the same approach as described above, first, the main effects of all predictors were entered, then the main effect of group was entered, and finally all interactions between predictors x group were entered. When assessing the first step of this analysis, the model was significant [*F*_change(3, 61)_ = 10.86, *p* < 0.001, *R*^2^ = 34.8%]. The main effects of each predictor in this model (BDI, CUDIT-R, and craving) showed that higher BDI (*B* = 0.401, *SE* = 0.14, *p* = 0.006) and higher CUDIT-R (*B* = 0.444, *SE* = 0.20, *p* = 0.028) scores at baseline were predictive of greater withdrawal at follow-up. Adding the main effect of group in a second step did not yield a better model fit (*p* = 0.10), as well adding all interactions between group and these predictors did not improve model fit (*p* = 0.277) in a final step.

### Control analyses

We performed two control analyses. First, to control for overlap between the MWQ symptoms Depression and Craving and our main predictors of interest BDI (Depression) and MCQ-SF (Craving) we repeated all regression analyses with a withdrawal (MWQ) score excluding these two items. All reported results and interpretations remained the same, except for the BDI. That is, BDI was a significant predictor for withdrawal severity [*F*_change__(1, 63)_ = 20.18, *p* < 0.001, *B* = 0.50, *SE* = 0.11] yet the interaction between group x BDI now reached significance [*F*_change__(1, 61)_ = 4.4, *p* = 0.04]. Follow-up analyses showed that BDI scores were more strongly related to withdrawal in the experimental group [*F*_change__(1, 27)_ = 12.3, *p* = 0.002, *B* = 0.714, *SE* = 0.20] than in the control group [*F*_change__(1, 34)_ = 4.77, *p* = 0.036, *B* = 0.248, *SE* = 0.11].

Second, nicotine dependence (FTND) correlated positively with cannabis use-related problems (CUDIT-R). To control for a possible influence of nicotine dependence, we repeated all regression analyses adding the FTND as an additional predictor in the first step, and the interaction between group x FTND as an additional predictor in the third step of the regression models. All reported results and interpretations remained the same, and no main or interaction effects of FTND were observed.

## Discussion

Not all individuals with a CUD who attempt to lower or quit their cannabis use will experience severe withdrawal (9). From a clinical perspective, predicting who will could help tailoring treatment (e.g., decisions to commence pharmacological and/or psychological withdrawal management). In this first study in near-daily cannabis users, we investigated if cognitive (craving, cognitive control) and mental health (depression, cannabis use-related problems) factors could predict withdrawal severity during an active attempt to cut down cannabis use. The experimental group was asked to lower substance use as much as possible in the week following the baseline test-session and significantly reduced their cannabis use more and experienced more withdrawal compared to the control group. Baseline cannabis use-related problems, depressive symptoms and craving, *but not cognitive control*, significantly predicted withdrawal at follow-up. Moreover, craving uniquely predicted withdrawal severity in the experimental group. Only depressive symptoms and cannabis-use-related problems uniquely predicted withdrawal severity across groups. Together, these factors explained 34.8% in the variance in cannabis withdrawal 1 week later. For the first time we showed that especially baseline depressive symptoms, cannabis use-related problems and craving, but not cognitive control, could be indicative of cannabis withdrawal. Severity of depressive symptoms and cannabis use-related problems may thereby predict general mental and physical discomfort from fluctuations in cannabis use, whereas craving may specifically do so in individuals who actively attempts to cut down cannabis use.

This study is a first step toward understanding the mechanisms underlying cannabis withdrawal during an active attempt to cut-down or quit cannabis use, generating important new hypotheses about how different self-reported mental health problems can uniquely contribute to self-reported cannabis withdrawal severity; Regardless of the significant moderate correlations between depressive symptoms and cannabis use-related problems, both factors uniquely predicted withdrawal severity across groups. Craving predicted withdrawal severity more strongly in the group who attempted to lower their cannabis use. This effect was no longer significant when depressive symptoms and cannabis use-related problems were entered into the regression model, indicating that the shared variance of these factors with craving is probably driving the interaction between group and craving. Importantly, to further our understanding, these results should be followed up by more in-depth pharmacological studies, including longitudinal measures of withdrawal and objective biochemical measures of cannabis, cigarette and alcohol use. A critical evaluation of the design and the implications for theory and future studies is outlined below.

A cannabis user is almost never only a cannabis user. This is corroborated in the present study; 90% of the cannabis users combined tobacco with cannabis in their joints, 72% of the cannabis users were daily cigarette smokers, and cannabis use-related problems correlated with nicotine dependence (*r* = 0.363) and depressive symptoms (*r* = 0.379). Alcohol use did not correlate with cannabis and cigarette use. Cannabis use was lower at follow-up and this decline was larger for the experimental group, indicating that participants indeed engaged in an active attempt to cut down. However, cigarette use was also slightly lower at follow-up independently of group. Cigarette use has been shown to predict cannabis dependence over time, independently from cannabis use frequency (45). Moreover, the pattern and timeline of cannabis and nicotine withdrawal is suggested to be similar and simultaneous abstinence may increase withdrawal symptom severity (46). These results raise the question if nicotine withdrawal and dependence are partly driving the results. Cannabis use-related problems and depressive symptoms uniquely predicted self-reported withdrawal severity after 1 week and these results did not change when nicotine dependence was additionally controlled for. Moreover, daily cigarette use did not correlate with daily cannabis use (FTND) and the change in cigarette use did not correlate with change in cannabis use and withdrawal severity. These observations suggest that nicotine withdrawal and dependence are not driving the result. However, studies investigating the biochemical interactions between cannabis and cigarette use and abstinence, and their potential detrimental effects on clinical outcomes are needed to further investigate this.

Following contemporary addiction models (16, 19–23) we hypothesized that individuals with good cognitive control would be better in suppressing withdrawal. Cognitive control did not predict cannabis withdrawal, in contrast to mental health factors and craving. These findings suggest that both relatively “cold” executive functioning (Stroop) and relatively “hot” decision making (CCT) are unrelated to withdrawal. The two control measures did not correlate with each other and any of the substance use measures in the sample that completed the entire study (*n* = 65). However, *post-hoc* analyses in the entire sample (*n* = 90) revealed a moderate correlation between Stroop and CCT (*r* = 0.364, *p* < 0.001) and weak correlations between craving and cognitive control (Craving-Stroop *r* = 0.242, *p* = 0.022; Craving-CCT *r* = 0.207, *p* = 0.05), supporting moderate construct and external validity of the CCT and Stroop. To the best of our knowledge, there are no studies directly investigating the association between cognitive control and cannabis withdrawal. Interestingly, a meta-analysis investigating the association between nicotine abstinence and cognitive performance suggests that response inhibition is consistently impaired by nicotine abstinence, but not cognitive control as measured with the Stroop (47). Moreover, an fMRI study on smoking abstinence suggested that particularly the regulation of negative affect was related to abstinence (31). These studies suggest that behavior and brain functioning during cognitive tasks that measure (negative) emotion regulation and inhibition may predicts cannabis withdrawal better than the Stroop and CCT. However this hypothesis is speculative and future studies are needed to further unravel the relationship between different aspects of cognitive functioning and withdrawal.

Cannabis withdrawal symptoms include, irritability, anger or aggression, depressive mood, anxiety, sleep difficulty (including strange dreams), loss of appetite, restlessness, and discomforting physical symptoms like tremors, headaches, sweating and abdominal pain (2, 15). Importantly, depressive mood and craving (on a 4-point scale) are symptoms of cannabis withdrawal, showing some overlap with the BDI and craving assessment, but these two items do not drive the results as the effects are still significant and even stronger for the BDI when the depressive mood and craving items are excluded from the analyses. Moreover, explorative analyses of individual withdrawal symptoms (Figure 1) indicated that the group difference in withdrawal was mainly driven by strange dreams irritability and decreased appetite. Unfortunately, we only assessed withdrawal once after 1 week (close to the expected average peak of withdrawal) (8, 13, 15). These results therefore reflect average severity of each symptom across the week following the baseline test-session. To assess the clinical merit of our results and to further investigate the association between mental health, craving and withdrawal it essential to study individual withdrawal symptoms over *the course* of abstinence in general population and clinical samples of cannabis users.

As discussed above, withdrawal was only assessed once. The current study also has other limitations that should be acknowledged. The most important limitation is that we did not include a biochemical verification of abstinence. In this first study, we opted for a simple design, testing an ecologically valid sample of cannabis users with comorbid substance use, using behavioral tasks and self-reports that are generally available to clinicians. Higher withdrawal combined with lower self-reported cannabis use in the experimental compared to the control group and the observed correlation between different mental health assessments suggest that the self-reports and procedures are meaningful. However, without objective measures of substance use we cannot rule out the potential effect of a social desirability bias induced by the testing procedure and cannot properly investigate the interactions between withdrawal, cannabis, cigarette and alcohol use. Although differentiating new cannabis use from residual cannabis metabolites in urine samples remains hard, statistical modeling tools using creatine and 11-nor-9-carboxy-Δ9-tetrahydrocannabinol (THCCOOH) levels showed promising results and can aid in future studies (48).

Moreover, the mental-health factors included in this study were limited to substance use-related problems and depression. We specifically choose to only include depressive symptoms, given that previous studies showed that only mood disorders were related to the experience of significant withdrawal in heavy adult cannabis users (14) and in treatment seeking adolescent cannabis users (7). Yet, other mental health factors may play a role withdrawal as well as both internalizing and externalizing disorders are highly comorbid with CUDs and differentiate between cannabis users with and without a CUD (49). Also, dropout in the current study was 28% and those who dropped out had significantly higher cannabis use-related problems than those who participated in the follow-up. It is possible that difficulty with decreasing cannabis use is related to the reachability of those participants. Since selection effects may drive the current findings, it is important for future studies to verify these findings across different populations. The unexpected difference between the groups on the CCT, should also be acknowledged. The experimental group took more risky decisions than the control group and this may have influenced our findings. Moreover, the relative limited sample size is a limitation and studies in older cannabis users are needed to determine of our effects generalize to older age-groups. Finally, only a limited number of females were tested, hindering us to assess potential differential impact of sex on withdrawal [(50), but see (9, 12, 51, 52)]. Taken the strengths and limitations of this study together, an important next step is to study the role of a wider set of cognitive and mental-health factors in individual trajectories of withdrawal, in a well-balanced sample of males and females, both inside and outside of a clinical setting, with the former having the advantage of monitoring a broader group of heavy cannabis users, and the latter having the advantage of monitoring cannabis abstinence and participation in a more controlled setting.

In conclusion, we showed that baseline depressive symptoms, cannabis use-related problems and craving, but not cognitive control, could be indicative of cannabis withdrawal during an attempt to cut down cannabis use. These findings generate new hypotheses about how mental health and cognitive factors may uniquely contribute to cannabis withdrawal. However, this study did not include biochemical verifications of abstinence and should be follow up by studies including more objective measures of substance use and more frequent assessments of cannabis withdrawal over time, both inside and outside of a clinical setting.

## Author contributions

JC and AvD equally contributed to the study design, data collection and data analysis. JC drafted the manuscript and AvD provided critical revisions. Both authors approved the final version of the manuscript.

### Conflict of interest statement

The authors declare that the research was conducted in the absence of any commercial or financial relationships that could be construed as a potential conflict of interest.
